# AutoCellANLS: An Automated Analysis System for Mycobacteria-Infected Cells Based on Unstained Micrograph

**DOI:** 10.3390/biom12020240

**Published:** 2022-02-01

**Authors:** Yan Zhuang, Xinzhuo Zhao, Zhongbing Huang, Lin Han, Ke Chen, Jiangli Lin

**Affiliations:** 1 Department of Biomedical Engineering, Sichuan University, Chengdu 610065, China; yan.zhuang@foxmail.com (Y.Z.); zbhuang@scu.edu.cn (Z.H.); gjzzcc@163.com (L.H.); 2 Department of Biomedical Engineering, Shenyang University of Technology, Shenyang 110870, China; zhaoxinzhuo@sut.edu.cn

**Keywords:** neural networks, unsupervised learning, cell detection, phase-contrast micrograph, infection classification, tuberculosis

## Abstract

The detection of Mycobacterium tuberculosis (Mtb) infection plays an important role in the control of tuberculosis (TB), one of the leading infectious diseases in the world. Recent advances in artificial intelligence-aided cellular image processing and analytical techniques have shown great promises in automated Mtb detection. However, current cell imaging protocols often involve costly and time-consuming fluorescence staining, which has become a major bottleneck for procedural automation. To solve this problem, we have developed a novel automated system (AutoCellANLS) for cell detection and the recognition of morphological features in the phase-contrast micrographs by using unsupervised machine learning (UML) approaches and deep convolutional neural networks (CNNs). The detection algorithm can adaptively and automatically detect single cells in the cell population by the improved level set segmentation model with the circular Hough transform (CHT). Besides, we have designed a Cell-net by using the transfer learning strategies (TLS) to classify the virulence-specific cellular morphological changes that would otherwise be indistinguishable to the naked eye. The novel system can simultaneously classify and segment microscopic images of the cell populations and achieve an average accuracy of 95.13% for cell detection, 95.94% for morphological classification, 94.87% for sensitivity, and 96.61% for specificity. AutoCellANLS is able to detect significant morphological differences between the infected and uninfected mammalian cells throughout the infection period (2 hpi/12 hpi/24 hpi). Besides, it has overcome the drawback of manual intervention and increased the accuracy by more than 11% compared to our previous work, which used AI-aided imaging analysis to detect mycobacterial infection in macrophages. AutoCellANLS is also efficient and versatile when tailored to different cell lines datasets (RAW264.7 and THP-1 cell). This proof-of concept study provides a novel venue to investigate bacterial pathogenesis at a macroscopic level and offers great promise in the diagnosis of bacterial infections.

## 1. Introduction

As the causative pathogen of tuberculosis disease (TB), *Mycobacteria tuberculosis* (Mtb) is one of the most dangerous bacteria for public health and causes millions of deaths worldwide [1,2]. The detection of Mtb is a critical step for global TB control. The most used strategy is to target Mtb specific antigens [3,4]. EsxA (6-kDa early secreted antigenic target, ESAT-6), a secreted substrate of the ESX-1 secretion system of Mtb, is an essential virulence factor of Mtb. EsxA is believed to mediate phagosome rupture and translocate Mtb to the cytosol of host cells [5,6]. Since EsxA is not present in BCG vaccine strain [7], it becomes an ideal biomarker for Mtb detection. Besides, as a T cell antigen, EsxA stimulates T cells to release interferon-γ [8]. Thus, the EsxA-based interferon-γ release assay was developed and widely used to detect Mtb infection [9]. Compared to the traditional way of culturing live Mtb organisms from patients’ sputum samples, which takes weeks to get results and has a low detection rate, infection-based molecular or immunologic assays usually take hours or days and have a higher detection rate. However, the detection of either EsxA gene or interferon-γ release assay cannot distinguish latent infection from active TB, which challenges their application as a detection method. Moreover, although the mycobacteria-infected mammalian cells could be detected by the fluorescence microscopy in live cells, it requires expensive instruments and time-consuming sample preparation. In contrast, the non-destructive phase contrast microscopy imaging has a lower cost compared with fluorescent images, greatly reduces phototoxicity, and simplifies sample preparation. Therefore, a novel, simple, and accurate method capable of detecting Mtb infection in phase contrast micrographs remains as an urgent need.

The existing cell analytical platforms, such as CellProfiler and ImageJ, etc., have limited features for the analysis of phase contrast images [10] due to the complex image features and difficulty of detecting cell populations. In the last few years, deep learning methods such as CNNs have shown remarkable performances for a wide range of computer vision tasks. Specifically related to classification problem, AlexNet [11], ResNet [12], and DenseNet [13] have achieved the state-of-the-art recognition level and have been adopted as the desired models for various medical imaging tasks [14,15,16]. Recently, numerous researchers have begun designing deep networks that are more suitable for their tasks [17]. There are few reports on the workflow of automatically detecting and analyzing morphological changes for each cell on the phase contrast microscopic images with dense cell distributions. As previously reported [18,19,20], CNN was first applied to the detection of mycobacteria-infected cells based on phase contrast micrograph to analyze and classify the morphological changes of the macrophages infected with Mycobacterium marinum (Mm), a surrogate model of Mtb. By using low-resolution phase contrast images, CNN could distinguish the infected cells from the uninfected cells at the early stage of infection, and the morphological changes are even indistinguishable for naked eyes. However, there are some limitations in the previous study [20]. Firstly, the cell detection algorithm lacks adaptability and requires human intervention due to the dense cell distribution on the microscopic images, which also leads to a lower accuracy. Secondly, the classification network based on Resnet 50 could not distinguish the cells infected with different Mm strains since these infections were very similar on the cell micrograph. Finally, the method was only tested in one cell line and needs to be verified on different cell lines.

In this paper, we tackle these difficulties and propose a novel deep learning system (AutoCellANLS) to automatically detect and analyze the macrophage cells infected by Mm in phase contrast images through image pre-processing, machine learning-based detection, and deep learning-based classification. The improved detection algorithm could adaptively and automatically segment cells in the cell population by combing the circular Hough transform (CHT) [21] with the Chan–Vese segmentation model [22,23], which overcomes manual intervention and improves the accuracy compared with the semi-supervised method [20]. Instead of using the existing network like Res-net50 [20], we designed a transfer learning enabled neural network (Cell-net) for cell classification with higher accuracy, efficiency, and versatility when it is applied to different cell line datasets.

The main contributions of our work are summarized as follows:

(1) We develop a novel system that automatically detects and analyzes the individual cells based on unstained phase-contrast images, which provides a novel pipeline to distinguish the infected cells that would be indistinguishable to the naked eye.
(2) We optimize the initial contour of the Chan–Vese model by circular Hough transform to achieve adaptive cell detection, which overcomes the shortcomings of manual setting parameters in previous studies.
(3) We establish an end-to-end architecture of a task-specific neural network by boosting the training of transfer learning for accurate classification with higher accuracy, efficiency, and versatility when applied to different cell line datasets.
(4) The experiments demonstrate that our method consistently achieves better results throughout the infection period (2 hpi/12 hpi/24 hpi) and increased the accuracy by more than 11% compared to several other state-of-the art methods.

The remainder of this paper arranged as follows. Section 2 introduces the main pipelines of our designed AutoCellANLS and describes the framework that is related to the proposed method. Section 3 presents the experiments and result analysis. Section 4 provides a discussion. Finally, Section 5 concludes our work.

## 2. Materials and Methods

### 2.1. AutoCellANLS

**Figure 1:**
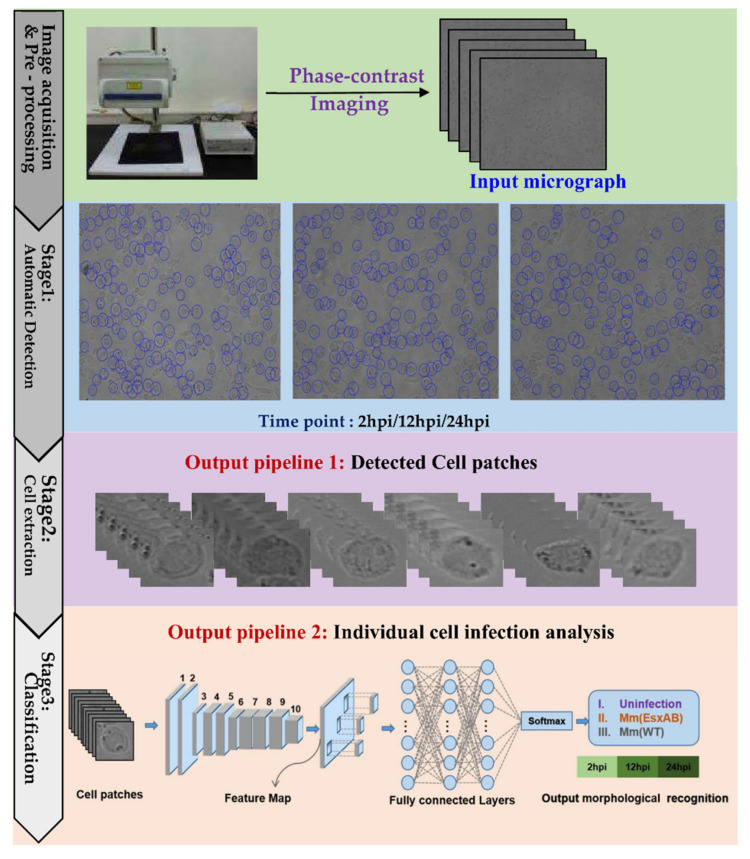
Overview of the proposed AutoCellANLS for infection analysis of different cell lines.

We have developed an automated AI framework for detecting morphological changes of the cells infected by mycobacteria without prior user training. The Figure 1 shows the user application pipelines of cell data analysis in the AutoCellANLS proposed in this paper. First, the cells were imaged in phase-contrast mode (whole cell morphology) as the input dataset fed into the workflow. In stage 1, the images were subjected to automated cell detection by machine learning algorithms (blue circles are the detection results). Following cell detection, the obtained single cell patches were the input dataset for classification, which included the uninfected cells and the cells infected by Mm(WT) and Mm(ΔEsxAB), respectively. In stage 3, the single cell datasets were fed into by our Cell-net to analyze the cells with intracellular mycobacterial infection. Finally, the result was used identify the morphological changes of the host cells at different times of post-infection (2 hpi/12 hpi/24 hpi). The whole analysis is based on the training of the machine learning algorithms with high flexibility, which can be tailored to different cell lines.

We demonstrate that AutoCellANLS enables the detection of cell morphology in microscopic images sensitively and accurately, without the need for extensive user interaction for data annotation. In addition, the automatic detection and deep learning of image-derived features overcome the dependence on user-curated feature analysis collections and accurate cell segmentation outlines. Hence, this application can accurately distinguish the infected cells from the uninfected cells by using the regular phase-contrast microscopic images. Moreover, it could further distinguish the morphological changes caused by Mm(WT) versus Mm(ΔEsxAB) strain, which correlates EsxA to specific morphologic features and demonstrates potential applications in TB diagnosis and research.

### 2.2. Cell Culture and Mycobacterial Infection

Mycobacterial strain and cell line, i.e., Mm(WT) and Mm(ΔEsxAB) strains, were reserved in the lab. To accurately identify infected cells, we labelled the two strains with mCherry-coding plasmid pMSP12::mCherry. For the infection assay, single cell preparations of the Mm strains were prepared as described elsewhere [24]. Briefly, Mm cells were first cultured in 7H9 media plus 10% oleic albumin dextrose catalase (OADC) and 0.05% Tween 80 at 30 °C. Then, the pellets were collected to pass through a 27-gauge syringe for several times to break the clumps. After that, the Mm cells were passed through a 5-nm filter for a further isolation. The colony forming unit (CFU) of single cell preparation was determined with a 10-fold series dilution method. The RAW264.7 and THP-1 cell lines were purchased from ATCC and reserved in the lab.

Infection assay of RAW264.7 and THP-1 cells—THP-1 cells were cultured in RPMI-1640 media with 10% FBS for three days. Then, THP-1 cells were plated into a 24-well plate at 2 × 105 cells per well and treated with 100 nM phorbol myristate acetate (PMA) overnight for differentiation. After that, PMA was removed, and the cells were rested for at least 48 h. For infection, single cell preparation of Mm(WT) and Mm(ΔEsxAB) were added into each well with 2 MOI. After a brief centrifugation, the cells were incubated at 30 °C for 4 h. The unbound bacteria were removed with three washes of 1 × phosphate saline buffer (PBS). Then, RPMI-1640 containing 100 mg/mL amikacin and 1% FBS were added and incubated for 1 h at 30 °C to kill the extracellular bacteria. The infected cells were cultured in RPMI-1640 containing 1% FBS and 50 mg/mL amikacin at 30 °C. At 2, 12, and 24 hpi, the cells were fixed by 4% paraformaldehyde and covered with 50% glycerol-water solution. The imaging of THP-1 cells was similar to that of RAW264.7 cells. Images were taken using a FLoid Cell Imaging Station (Life technologies) with a fixed 460× magnification. The images with the channels of bright field and red fluorescence (mCherry) were exported as 16-bit tagged image file format (TIFF) with a resolution of 96 dpi. The uninfected cells were treated exactly the same as infected cells and used as the control group. For RAW264.7 cells, the images taken in our previous study were again used for this study [20].

### 2.3. The Dataset

We used two cell lines for the experiments, namely human macrophage cell line THP-1 and mouse macrophage cell line RAW264.7. The cells of each cell line were divided into three groups. Thus, one group was uninfected as the control, and the other two groups were infected with either Mm(WT) or Mm(EsxAB). At 2, 12, and 24 h of post-infection, the cells were imaged under a fluorescence microscope with a red fluorescence channel for mCherry-expressing mycobacteria as the labels and a phase-contrast channel for cell morphology. For each cell line, the obtained cell micrograph dataset consisted of three groups for each time point post-infection (2 hpi/12 hpi/24 hpi), of which 60 were uninfected, 90 were infected with Mm(EsxAB), and 90 were infected with Mm(WT), for a total of 720 micrographs. After the cell detection, small amounts of cells are the infected cells at the instance-level (i.e., single cell patches). Instead of classifying the cells by the original image-level labels, it is preferred to distinguish which single cell is infected because in the images of the infected group, not all the cells are infected. Then, these single cells are used to train the CNN classifier to discriminate the true infected cells from the uninfected cells. For the THP-1 cell line, the number of cell patches utilized for classification is shown in Table 1.

### 2.4. Proposed Method

**Figure 2:**
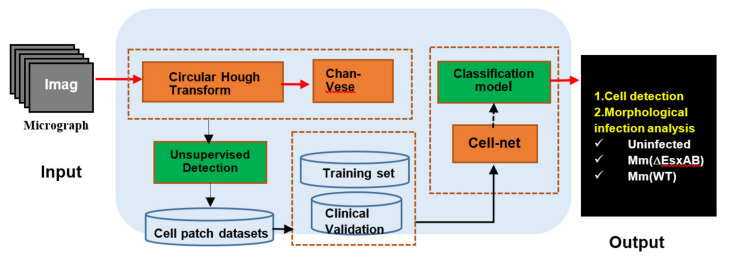
The diagram of algorithms and data in the AutoCellANLS.

To build the AutoCellANLS workflow, we developed a novel automatic detection method for cell segmentation first, which combines the circular Hough transform (CHT) [21] with the level set model (C-V) [23] to extract single cells from the cell population in the phase-contrast images. Afterwards, the detected cells were fed into to our Cell-net for further cell analysis. The whole method scheme was shown in Figure 2. Firstly, the phase-contrast micrograph dataset was input into the AutoCellANLS. Then, the single cell patches could be automatically extracted from the cell populations through an improved detection algorithm. To achieve automatic detection and classification, the cell patches dataset for each cell line was randomly divided into the training and validation sets based on cross-validation and fed into the deep learning module based on Cell-net for feature extraction and classification.

#### 2.4.1. Unsupervised Cell Detection

The circular Hough transform (CHT) algorithm is a feature-based learning method and does not need labeled input data, but it needs manual input of multiple parameters, such as the radius of the detecting circle [21], which presents limitations for detecting the complete cell area adaptively. Although the level set demonstrates good performance in image segmentation based on automatic iteration, it requires the manual input of parameters, such as the initial contour of the target [25]. Therefore, we improved the detection method based on our previous work [20] through combining the circular Hough transform and the Chan–Vese model to extract the contour of each cell without user intervention, which is unsupervised and improves the accuracy of the cell segmentation. When detecting circles, the points in the images are transformed to the parameter space, seen as Equation (1):

(1) $$r_{}{}^{2}={\left(x-a\right)}^{2}+{\left(y-b\right)}^{2}$$

Each point in the image is represented by three parameters: *r*, *a,* and *b*, where *r* is the radius of each detected circle, and *a* and *b* present the location of the center of the circle. Firstly, the gradient vector of the pixel at the position (*i,j*) is calculated by Equation (2):

(2) $$\nabla I_{i,j}=(v_{x},v_{y}){|}_{i,j}=(I_{i,j}-I_{i,j-1},I_{i,j}-I_{i-1,j})$$

where (*i*,*j*) indices the pixel position. *I_i,j_* is the intensity of the pixel. Then, an accumulation step is introduced to find the highest response of the gradient, which would be the center of the circle, and *q (i*,*j)* is the radial vector from the circle center to the pixel (*i*,*j*). The radius was defined as Equation (3), where ∆*r* is the interval between adjacent *r* values.

(3) $$\left|\|q\left(i,j\right)\|-r\right|<\Delta r/2$$

To ensure that only the cell was export to subsequent network and improve the accuracy of the final classification, the rough ROI from the circular Hough transform, which enclosed the cell region. According to the initial contour detected by circular Hough transform, a typical C–V level sets methodology [23] was applied to refine the cell boundary, which provides a complete outline for single cell patch extraction.

(4) $$E(C)=\mu_{1}{\int_{inside(C)}\left|I(x,y)-c_{1}\right|}^{2}dxdy+\mu_{2}{\int_{outside(C)}\left|I(x,y)-c_{2}\right|}^{2}dxdy+\alpha \kappa$$

where *I* is the image, *C* refers to the boundary of the segmented region, *c_1_* and *c_2_* are the respective averages of *I* inside and outside *C*, and *κ* is the curvature of *C*.

**Figure 3:**
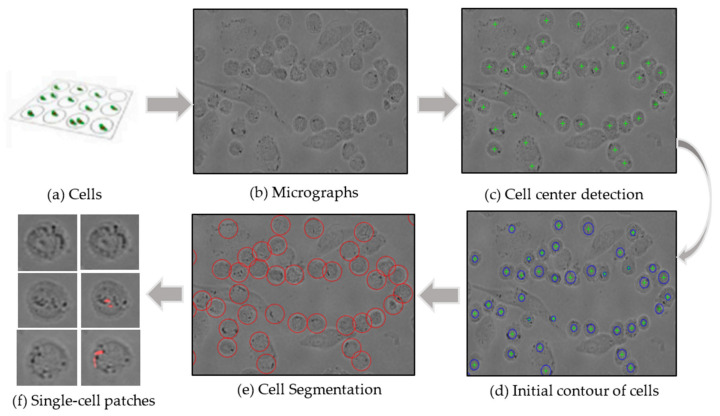
The Automatic detection process for THP-1 cell images.

In the previous study (Bao, et al.) [20], the Circular Hough Transform could be well positioned to the center of each cell, but the complete contour of each cell could not be detected because it requires manual setting of cell radius. Here, we improved the C-V model to optimize automatic segmentation based on the initial contour detected by Circular Hough Transform (Figure 3c. green crosses). As shown in Figure 3, the unsupervised detection in the cell population is divided into four processes that could detect each cell accurately and adaptively without manual selection of parameters. Firstly, the circular Hough transform algorithm is used to obtain the suggested cell regions in the images (Figure 3c). Second, the detected circle of each cell region is extracted based on the circular Hough transform algorithm as the initial contour (Figure 3d), which may not accurately find the complete cell boundary because the cell radius needs to be set manually. Third, we used the C-V segmentation model based on the initial contour in order to detect each cell more accurately and completely (Figure 3e). Finally, the single cell patches (the left column of Figure 3f) were automatically extracted from the regions of interest (ROI) in the phase-contrast images and screened into the Cell-net classified as uninfected cells (first row of Figure 3f), Mm(ΔEsxAB)-infected cells (second row of Figure 3f), and Mm(WT)-infected cells (third row of Figure 3f). The right column of Figure 3f shows the corresponding ground truth after fluorescence staining. The optimized algorithm has been validated on multiple cell lines, RAW264.7, A549, and THP-1, demonstrating its robustness, effectiveness, and efficiency.

#### 2.4.2. Data Augmentation

The cells in the control group are automatically extracted as the input dataset of uninfected cells, while for the infection groups, only the cells containing the mCherry-expressing bacteria either within or on the circles are considered as infected cells and extracted from the images. Thus, we used image augmentation technology to optimize the classification process for cell datasets due to the rate of infection always being less than 100%, as shown in Table 2. The cell dataset was randomly split into the training dataset and validation dataset of THP-1 cells using 10-fold cross-validation. The quality of small data is improved by applying data augmentation techniques to the training dataset, which can effectively reduce the problem of overfitting.

#### 2.4.3. The Transfer Learning Enabled Cell-Net

A weight sharing mechanism in deep convolutional neural networks can drastically decrease the number of parameters and reduce complexity. The end-to-end architectures have more advantages when the data are multidimensional, so we can input images as parameters to the network directly. Compared with traditional feature extraction methods, this method is not hindered by complex feature extraction and data reconstruction.

**Figure 4:**
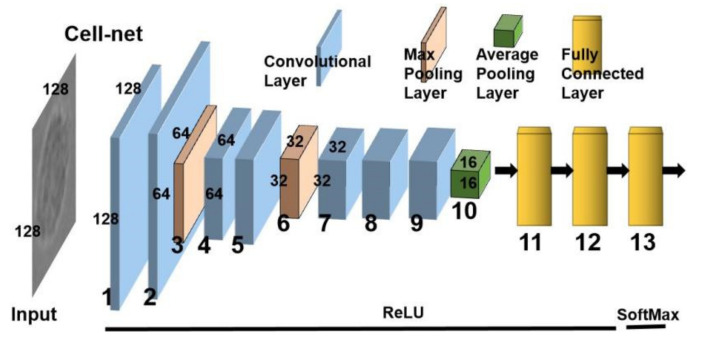
The structure of the Cell-net.

The Cell-net designed in this study uses several feature extraction stages, shown in Figure 4. The designed network is based on the characteristics of the cell images and contains seven convolutional layers (1, 2, 4, 5, 7, 8, and 9) and three pooling layers (3, 6, and 10) for extracting feature maps. Figure 4 shows the Cell-net diagram representing convolutional filters with respective width and height designated on filters facets. The size of the convolution kernel is 3 *3 with the one stride for each convolutional layer. The activation functions are depicted below the diagram. A parameter sharing scheme is used in convolutional layers to control the number of parameters while retaining the important features, including scale invariance, called invariance, rotation invariance, and translation invariance [26,27]. This model can effectively reduce overfitting and improve the DCNN generalization ability. For the pooling layers, we use the max pooling and global average pooling, which reduce the number of parameters. Commonly, the sets of weights are a filter (or a kernel) of the mean patch (average pooling, maximum pooling), whenever weights are not modified by back propagation. Image deformation influence can be weakened by this layer (e.g., translation, scale, and rotation). This decreases the number of parameters and improves model accuracy.

Then, the cells in the dataset at different periods (2 hpi/12 hpi/24 hpi) are divided into three categories (uninfected, Mm(EsxAB)-infected, Mm(WT)-infected) through two fully connected layers and a SoftMax layer. The accuracy and efficiency of this network in cell classification far exceed the Resnet50 used in the earlier study [20]. In addition, imbalanced data represents one of the challenges of machine learning, which affects the accuracy of classifiers [28]. Thus, the loss function is improved as focal-loss [29] to solve the imbalance of each cell dataset. The classification error $L_{cls}$ is defined as follows:

(5) $$L_{cls}=-\underset{c\in classes}{\sum }\left[\hat{P_{i}}\left(c\right)log\left(P_{i}\left(c\right)\right)+\left(1-\hat{P_{i}}\left(c\right)\right)log\left(1-P_{i}\left(c\right)\right)\right]\;$$

where *c* refers to the class (*classes* = 2) to which the detected cell patch belongs. $P_{i}\left(c\right)$ refers to the predicted value that the *i*-th cell patch belonging to class c, and $\hat{P_{i}}\left(c\right)$ is the ground truth.

According to transfer learning [30], we can fine-tune all the layers, including the convolutional layers. This means that all the weights inherited from the trained deep learning model are fine-tuned by the new task datasets and all layers participate in the backward computation. Besides, we used ImageNet [14] as initial weights in our network, which improves classification efficiency and accuracy. The first layer defines the images as input data and creates an image input layer of the same size as the training images. The following layers are the convolution and sub-sampling layers. By minimizing the amount of data to obtain meaningful information, the previous layer neuron represents the enhanced signal after removing noises. Then, the useful features are extracted and selected by a fully connected layer that converts 2D features into 1-D, which measures various classifier criteria. Finally, the classifier identifies cell images based on their characteristics and classifies them into different types.

## 3. Results

We have developed an automated AI framework for detecting morphological changes of the cells infected by mycobacteria without prior user training. Especially, two core technologies, namely an automatic detection algorithm and novel deep convolution network, were applied to the cell classification task. This yielded an accurate classification boundary for the subsequent classification of any morphological deviation in large-scale screening data. The AutoCellANLS was designed for rapid cell detection and morphology classification based on phase-contrast images, and its adaptability, accuracy, and efficiency were verified with different cell line datasets. We implemented the experiment on Win10 with a GTX 1080 Ti GPU, and the framework was implemented in MATLAB R2019b and Torch 0.4.1.

### 3.1. Cell Detection

To evaluate the detection performance, quantitative assessment was implemented among the detected cell patches. The undetected single cells are counted on the original image, named as false negative (FN). Some cells are correctly detected as true positive (TP). Besides those, some non-cell regions are detected as cells, named as false positive (FP). According to these evaluation parameters, the accuracy, precision, and sensitivity are calculated, respectively, as shown in Table 3. According to the detection results, we noticed that the novel cell detection method could effectively detect the uninfected cells and the cells infected with either Mm(WT) or Mm(ΔEsxAB), which demonstrates the application prospects in infected cell screening among other cell lines in phase-contrast images. In the automatic detection stage, the non-cellular area may be mis-detected by the circular Hough transform or the under-segmentation of the cell area, which will be modified through the screening of the C-V model. Thus, the non-cell regions were not detected as cells and the accuracy was improved. In addition, the improved algorithm makes the detected cell edges more complete, which improves the accuracy of cell detection and neural network-based cell infection analysis. Since the infection rate is often less than 100%, meaning not all of the cells in the infection groups of cell micrographs are infected, we filtered out the uninfected cells using an extra image processing step to select the cell datasets for classification in the next stage.

### 3.2. Morphology Classification

**Figure 5:**
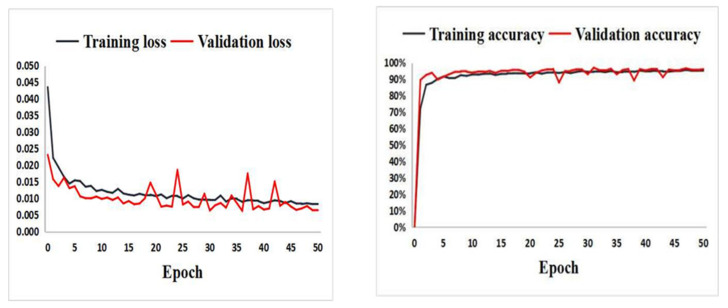
The accuracy and loss for the groups of the uninfected cells and the Mm(WT)-infected cells at 24 hpi in the THP-1 dataset.

With the designed Cell-net structure, data augmentation, and transfer learning strategy, the extracted single cells were used to tune the network. The training loss and the validation accuracy at 24 hpi are presented in Figure 5. With the increase of epoch numbers, the training loss gradually decreased, while the validation accuracy quickly reached the maximum at the early epochs and almost remained stable through all epochs, reaching 95% average accuracy, in turn indicating that the Cell-net is stable and effective.

**Figure 6:**
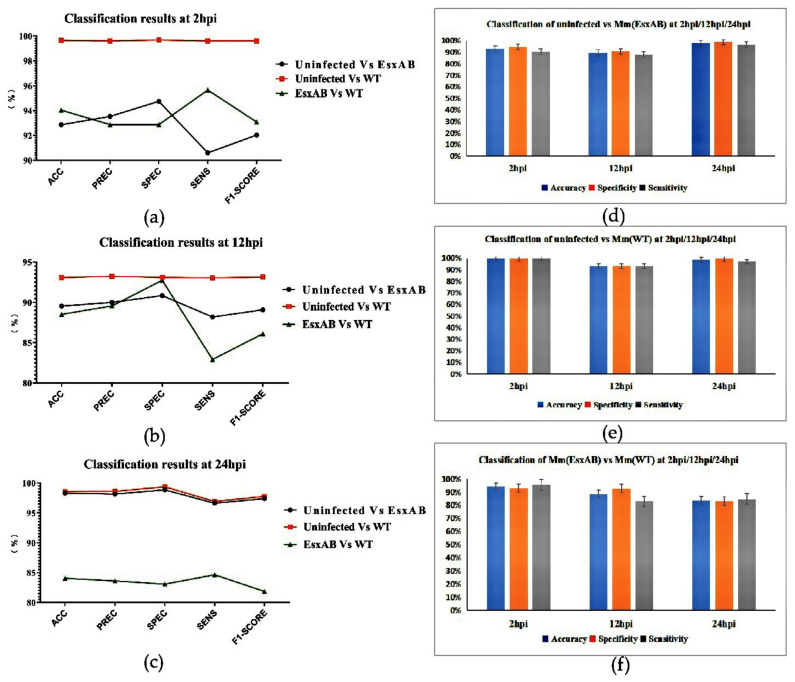
Infection analysis results of THP-1 cell dataset through the AutoCellANLS (The cell classification results were evaluated by accuracy, precision, specificity, sensitivity and F1-Score. (**a**–**c**) show the results for different cell groups (uninfected vs. Mm(ΔEsxAB), uninfected vs. Mm(WT), and Mm(ΔEsxAB) vs. Mm(WT)) at the same time periods, respectively. In addition, we also compared the classification results (**d**–**f**) for the same group of cells in different periods (2 hpi/12 hpi/24 hpi) to obtain the morphological changes over time during the process of cell infection. (ACC: accuracy, (TP + TN)/(TP + TN + FP + FN); PREC: Precision, TP/(TP + FP); SPEC: Specificity, TN/(FP + TN); SENS: Sensitivity (or Recall), TP/(TP + FN); F1-Score: (2 × Precision × Recall)/(Precision + Recall)).

To further validate the performance of our network, the classification results were assessed by accuracy score, numbers of true positive (TP), false positive (FP), true negative (TN), and false negative(FN) as well as F1-score (Figure 6). The single cell patches from the THP-1 cell dataset were divided into three binary groups (Uninfected/Mm(ΔEsxAB), Uninfected/Mm(WT), Mm(ΔEsxAB)/Mm(WT) at different times of post-infection (2/12/24 hpi). The classification results of the Uninfected/Mm(WT) groups at the 2/12/24 hpi show the best performance among the three groups for all the evaluation parameters (accuracy, precision, specificity, sensitivity, F1-score), which reached as high as 99% at 2 and 24 hpi. This indicates our fine-tuned network accurately distinguishes the WT-Mm infected THP-1 from the uninfected cells. At 2 hpi (Figure 6a), the classification results of the uninfected/Mm(WT) groups were the best with all parameters reached more than 99%. For classification of uninfected/Mm(ΔEsxAB), its accuracy, sensitivity and F1-score were the lowest. Moreover, its precision and specificity were a little higher than that of Mm(ΔEsxAB)/Mm(WT). For the classification of Mm(ΔEsxAB)/Mm(WT), its accuracy, sensitivity, and F1-score were between uninfected/Mm(WT) and uninfected/Mm(ΔEsxAB) pairs, while the other parameters were the lowest at this time point. This result indicates that at the early stage of infection, WT-Mm induce morphological changes of THP-1 that could be effectively detected by the Cell-net. For Mm(ΔEsxAB), the lower detection accuracy and sensitivity might be attributed to lack of EsxA and EsxB, but the infection still induces morphological changes for Cell-net detection. It is reasonable for the classification of Mm(ΔEsxAB)/Mm(WT) to exhibit a middle accuracy and sensitivity. At 12 hpi (Figure 6b), the specificity for uninfected/Mm(ΔEsxAB) pair (90.84%) is lower than that of the uninfected/Mm(WT) pair (93.10%) and Mm(ΔEsxAB) /Mm(WT) pair (92.74%), respectively. Meanwhile, the difference in sensitivity was more variant, and the classification results of Mm(ΔEsxAB)/Mm(WT) pair was the lowest (82.92%) compared with uninfected/Mm(ΔEsxAB) pair (90.84%) and uninfected/Mm(WT) pair (93.10%). regarding the general performance of Cell-net at this time point, it was weaker than that of 2 hpi, which indicates that Cell-net detect THP-1 cell’s morphological changes by Mm infection with lower efficiency at this time point. Interestingly, at 24 hpi, the classification results for all evaluation parameters between the uninfected cells and the other two types of infected cells were basically the same, and both were as high as 98% on average. For Mm(ΔEsxAB)/Mm(WT) pair, the parameters were all between 82% and 84%, which are much lower than the other two classification pairs. This indicates that at 24 hpi, the infection of Mm(WT) or Mm(ΔEsxAB) induced similar morphological changes in THP-1 cells. According to Cell-net’s performance at all three time points, we can conclude that our Cell-net could effectively detect THP-1 cells infected with Mm.

We have illustrated our results for THP-1 cells for each group in Figure 6. At 2, 12, and 24 hpi, the accuracy of detection between the uninfected cells and the Mm(WT)-infected cells was 99.65%, 93.07%, and 98.56%, respectively (Figure 6e), suggesting that our Cell-net was able to accurately distinguish the Mm(WT)-infected cells from the uninfected cells all the time. Moreover, the accuracy of our network to detect Mm(EsxAB)-infected cells from the uninfected cells was 92.86%, 89.55%, and 98.29%, respectively (Figure 6d), especially with the overall highest accuracy at 24 hpi. Meanwhile, the accuracy of detection between the cells infected with Mm(WT) or Mm(EsxAB) was much lower at all hpi points, with 94.04%, 88.52%, and 84.05%, respectively (Figure 6f). The specificity for all the detections reached more than 90%, except for the one between the Mm(WT) cells and Mm(EsxAB)-infected cells at 24 hpi (83.07%) (Figure 6f). The sensitivity of the classification exhibited a greater variation, in which the uninfected and Mm(WT)/Mm(EsxAB) groups demonstrated the highest sensitivity at more than 90% at all time points, while the Mm(WT)/Mm(EsxAB) group showed the lower sensitivity at 12 hpi with 82.92% and 24 hpi with 84.64%.

## 4. Discussion

The present study has shown the proposed AutoCellANLS for the detection and identification of morphological changes in cells infected by mycobacteria in phase-contrast images. As shown in Figure 6, the accuracy of detection varies at different times of post infection. Interestingly, the accuracy at 12 hpi, being significantly lower than that at 2 and 24 hpi (Figure 6d,e), indicates that the infected cells make more dramatic morphological changes at 2 and 24 hpi than 12 hpi. In fact, the CNN-detected dynamic morphological changes in response to mycobacterial infection are well correlated to many of the reported studies on host–pathogen interactions. For instance, it is well known that macrophages demonstrate spontaneous and strong immune responses at the very early stage of infection after encountering pathogens [31,32]. Thus, it is not surprising that CNN can accurately detect the morphological changes at 2 hpi. After being internalized into the macrophages, mycobacteria have been shown to stay latent within the phagosomes and suppress the host immune responses, which usually take place within 24 hpi [33,34,35]. Thus, the macrophages at 12 hpi stage show reduced morphological changes, supporting the observed lower detection accuracy at this stage. Thus, our method could accurately detect significant morphological changes between the infected and the uninfected cells in 2 hpi/12 hpi/24 hpi based on the micrograph of THP-1 cell line.

In order to further verify the capability of this approach for analyzing large-scale phase-contrast images, we used another cell line image dataset (RAW264.7) and performed our optimized AutoCellANLS for automated analysis workflow. RAW264.7 cell line, a type of macrophage cell, is commonly used for TB research. Similarly, the RAW264.7 cells were infected with Mm(WT) and Mm(EsxAB), followed by our system analysis. The classification result shows that the accuracy, sensitivity, and specificity of each group are improved a lot compared with our previous result [20]. Notably, in the classification of the uninfected/Mm(EsxAB) group, the accuracy rate increased from less than 80% to more than 93%, while in the uninfected uninfected/Mm(WT) group the accuracy improved from the highest, 90%, at 2 hpi and the lowest, 80%, at 12 hpi to an average of 95% at 2, 12, and 24 hpi. Besides, we compared the detection methods based on the RAW264.7 cell line, as shown in Table 4. The experimental results demonstrate that our method is especially helpful when the system must learn from cell populations and reduce the time of analysis for infected cells while enhancing the prediction accuracy and stability at the same time. This is often the case in medical settings, where data collection is either expensive (due to the requirement of highly trained experts) or time-consuming (due to the multiple repetitions of an experiment). In addition, the ability to generalize well with our datasets makes the proposed artificial workflow an appealing choice for clinical domains with large images or volumetric data.

The advantages of the proposed AutoCellANLS run through the entire process of cell detection and infection analysis compared with a previous study [20]. In the cell detection stage, we designed an automatic detection method by combining the circular Hough transform with the Chan–Vese segmentation model, which overcomes the drawbacks of semi-supervised models, such as manually parameter setting. Furthermore, the Chan–Vese model was used to finely detect the boundary of each cell, which solved the problem of the missed detection of irregularly shaped cells that occurred in our previous study [20]. Therefore, our unsupervised automatic cell detection method not only reached higher accuracy, but also avoided the time-consuming manual labeling of cell segmentation compared to supervised U-net [36], which is a state-of-the-art architecture for the detection and segmentation of cells in light microscopy images. In the stage of the infection morphology analysis, the proposed Cell-net aimed to achieve higher classification accuracy with fewer layers to avoid too many parameters for the robustness reduction of the network. In addition, we used some transfer learning strategies. For example, we utilized ImageNet [14] as the initial weights in our network to improve classification efficiency and accuracy. In order to minimize the impact of data imbalance, we improved the loss function and applied data augmentation technology. Thus, the proposed Cell-net showed a good performance with more robustness, effectiveness, and preciseness for cell classification when applied to different cell lines compared with the existing networks [36,37,38,39], such as ResNet [20], Inception_V3, and Xception, as shown in Table 5.

In summary, our automated AutoCellANLS could effectively detect and classify Mm infection in both human macrophage THP-1 cells and murine macrophage RAW264.7 cells based on cell micrographs. AutoCellANLS is a promising application to detect bacterial infection in phase-contrast images without other staining procedures. Similar to the results previously acquired from murine macrophage RAW264.7 in THP-1 cells, Cell-net’s performance in infection detection at 2 and 24 hpi is better than that at 2 hpi, which indicates a dynamic status of THP-1 morphological changes induced by Mm infection. Unlike the previous results, for RAW264.7 cells, the automated AI workflow distinguished either Mm(ΔEsxAB) or Mm(WT) from the uninfected cells effectively at all time points, indicating this workflow is more sensitive to morphological changes on infected RAW264.7 cells. Alternatively, THP-1 and RAW264.7 have different responses to Mm infection. For classification between Mm(ΔEsxAB) and Mm(WT), the automated AI workflow’s performance was generally weaker in both cell lines. Considering its better performance in infection detection and the dramatic virulence difference between Mm(ΔEsxAB)/Mm(WT) strains, we assume that the AI workflow has lower sensitivity to morphological changes, especially to EsxA and EsxB protein. By testing on different cell line datasets, our workflow can not only detect cells in the cell population with higher accuracy without manual intervention, but also identify morphological changes in individual cells through the designed Cell-net. This workflow greatly improves the accuracy, adaptability, and efficiency during the entire process for cell infection analysis. We expect the proposed infection analysis classifier to have high clinical significance. Consistent with prior studies on the tracking of individual cells within the series of phase-contrast images, an accuracy of classification from 90% to 95% in every single image is essential for biological users to accept the automated results without the need for manual proof-reading and editing [40,41].

## 5. Conclusions

The proposed AutoCellANLS has been presented as an automated pipeline for the detection and identification of morphological changes in cells infected by mycobacteria in the phase-contrast images, which is a promising application to detect bacterial infection in phase-contrast images without other staining procedures. It is essential to know where those changes are located and what cellular structures are affected, which will provide clues in future studies to reveal the in-depth mechanism of host–pathogen interaction. In addition, the experimental results demonstrate that our method is especially applicable when the system must learn from cell populations and reduce the time of analysis for infected cells while enhancing the prediction accuracy and stability at the same time. This is often the case in medical settings, where data collection is either expensive (due to the requirement of highly trained experts) or time-consuming (due to the multiple repetitions of an experiment). Therefore, the ability to generalize well with our datasets makes the proposed artificial workflow an appealing choice for clinical domains with large images or volumetric data.

## Figures and Tables

**Table 1:** THP-1 cell dataset for Cell-net classification.

	Date	Cell Type	No. of Cells	Total
Time				
2 hpi		uninfected	2782	7292
		Mm(ΔEsxAB)	2324	
		Mm(WT)	2186	
12 hpi		uninfected	3616	10,004
		Mm(ΔEsxAB)	3378	
		Mm(WT)	3010	
24 hpi		uninfected	4067	11,109
		Mm(ΔEsxAB)	3798	
		Mm(WT)	3244	

**Table 2:** Parameters setting of data augmentation.

Parameters	Range	Description
rotation_range	0.2	rotation range
rotation_scale	1/255	ratio of image magnification
shear_range	0.2	range of projection transformation
zoom_range	0.2	ratio of randomly zooming image
horizontal_flip	1	range of horizontal translation

**Table 3:** The detection results of Auto-AISDC for THP-1 cells.

	Image Type	No. of Microscopy	Detection Results				
			No. of Single Cell		Accuracy	Precision	Sensitivity
2 hpi	Uninfected	60	5746	19,236	94.63%	99.61%	94.99%
	Mm(ΔEsxAB)	90	8274				
	Mm(Wt.)	90	5216				
12 hpi	Uninfected	60	5802	25,267	95.05%	99.79%	95.24%
	Mm(ΔEsxAB)	90	9958				
	Mm(WT)	90	9507				
24 hpi	Uninfected	60	7687	29,206	95.72%	99.82%	95.88%
	Mm(ΔEsxAB)	90	10,934				
	Mm(WT)	90	10,585				

**Table 4:** The performance of the proposed AutoCellANLS on RAW264.7 cell dataset.

	RAW264.7 Cell Line	Results Evaluation				
		Accuracy	Precision	Specificity	Sensitivity	F1-Score
2 hpi	Uninfected vs. Mm(ΔEsxAB)	93.32%	95.87%	97.03%	88.56%	92.07%
	Uninfected vs. Mm(WT)	96.96%	97.95%	98.31%	95.35%	96.63%
	Mm(ΔEsxAB) vs. Mm(WT)	90.70%	90.70%	90.70%	90.70%	90.70%
12 hpi	Uninfected vs. Mm(ΔEsxAB)	97.79%	96.50%	96.32%	99.23%	97.85%
	Uninfected vs. Mm(WT)	95.58%	96.36%	96.49%	94.64%	95.50%
	Mm(ΔEsxAB) vs. Mm(WT)	84.80%	83.41%	84.36%	85.27%	84.33%
24 hpi	Uninfected vs. Mm(ΔEsxAB)	97.18%	97.57%	97.57%	96.79%	97.18%
	Uninfected vs. Mm(WT)	97.94%	96.99%	97.03%	98.89%	97.93%
	Mm(ΔEsxAB) vs. Mm(WT)	88.54%	93.10%	94.12%	82.73%	87.61%

**Table 5:** The Comparison of THP-1 cell classification results for different methods.

	THP-1 Cell Line	Accuracy			
		Resnet_50	Inception_V3	Xception	AutoCellANLS
2 hpi	Uninfected vs. Mm(ΔEsxAB)	75.17%	76.01%	74.76%	92.86%
	Uninfected vs. Mm(WT)	84.16%	83.20%	83.36%	99.65%
	Mm(ΔEsxAB) vs. Mm(WT)	71.26%	68.27%	70.10%	94.04%
12 hpi	Uninfected vs. Mm(ΔEsxAB)	68.39%	67.90%	68.47%	89.55%
	Uninfected vs. Mm(WT)	80.00%	76.02%	77.39%	93.07%
	Mm(ΔEsxAB) vs. Mm(WT)	70.87%	68.35%	68.83%	88.52%
24 hpi	Uninfected vs. Mm(ΔEsxAB)	85.32%	83.88%	84.48%	97.99%
	Uninfected vs. Mm(WT)	91.09%	89.30%	90.77%	98.56%
	Mm(ΔEsxAB) vs. Mm(WT)	72.11%	70.92%	73.23%	83.75%
